# The prognostic significance of CCL2 in oral squamous cell carcinoma: insights from tissue expression and perioperative serum profiling

**DOI:** 10.3389/fonc.2026.1764317

**Published:** 2026-03-27

**Authors:** Senli Wen, Haolei Tan

**Affiliations:** 1 The First Department of Head and Neck Surgery, Hunan Cancer Hospital, the Affiliated Cancer Hospital of Xiangya School of Medicine, Central South University, Changsha, Hunan, China; 2 Department of Breast and Thyroid Surgery, The First Hospital of Changsha, the Affiliated Changsha Hospital of Xiangya School of Medicine, Central South University, Changsha, Hunan, China

**Keywords:** CCL2, lymph node metastasis, oral squamous cell carcinoma, perioperative monitoring, prognosis, serum biomarker

## Abstract

**Objective:**

This study aims to examine the expression patterns of chemokine CCL2 in oral squamous cell carcinoma tissues, adjacent tissues and tumor microenvironment, including the examine of the paraffin block and the examination of the peripheral blood. It seeks to analyze the associations between CCL2 expression and clinical features, pathological characteristics, and prognosis, as well as assess its potential as a prognostic biomarker.

**Methods:**

We enrolled 119 patients with OSCC in this study. Immunohistochemistry detected CCL2 protein expression in cancerous and adjacent tissues. We used enzyme-linked immunosorbent assay (ELISA) to quantify serum CCL2 concentrations in patients both pre- and post-surgery. The chi-square test analyzed the correlation between CCL2 expression and clinical characteristics. We applied the paired t-test to compare pre- and post-surgery CCL2 changes. The Kaplan-Meier method evaluated CCL2’s impact on overall survival (OS) and disease-free survival (DFS) of patients.

**Results:**

Immunohistochemical analysis revealed that CCL2 expression was significantly elevated in OSCC tissues compared to adjacent normal tissues (P < 0.05). High CCL2 expression correlated strongly with advanced T stage (P = 0.002), clinical stage (P = 0.001), and lymph node metastasis (P = 0.001). Survival analysis showed that patients with elevated CCL2 levels in tumor tissues had significantly shorter overall survival (P < 0.001). Serological analysis indicated a significant overall decrease in postoperative serum CCL2 levels (P = 0.002); however, this decrease was not pronounced in patients with advanced stages (T3-4, stage III-IV) or recurrence. A combined analysis revealed that patients with high CCL2 expression in OSCC and increased postoperative serum CCL2 had the poorest disease-free and overall survival (P < 0.05). Conversely, patients with high CCL2 expression in OSCC and decreased postoperative serum CCL2 exhibited the best disease-free and overall survival (P < 0.05).

**Conclusion:**

The expression level of chemokine CCL2 in oral squamous cell carcinoma was significantly correlated with strong tumor invasiveness and poor prognosis. Persistently high CCL2 expression after surgery indicated a high risk of recurrence and metastasis, as well as poor prognosis. Dynamic monitoring of serum CCL2 levels is expected to become a biomarker for evaluating surgical efficacy and predicting recurrence risk. The CCL2/CCR2 signaling axis offers a new research direction for treating oral squamous cell carcinoma.

## 1 Introduction

Oral squamous cell carcinoma (OSCC) represents the most common form of oral cancer, comprising 90% of cases (1, 2). Research indicates that in advanced oral cancer, the metastasis rate to neck lymph nodes reaches 60% to 80%. Additionally, 20% to 40% of early-stage oral cancer patients experience occult neck lymph node metastasis (3). This relatively high rate of lymph node metastasis is the main cause of recurrence and has a significant impact on the prognosis of patients (4–6). Despite considerable advancements in basic research, clinical diagnosis, and treatment of head and neck squamous cell carcinoma in recent years, the five-year survival rate has persistently hovered around 50% (7, 8). Tumor invasion and metastasis involve more than just the actions of tumor cells (9). The interactions between tumor cells, the local cellular microenvironment, and the organism’s internal environment allow tumor cells to evade immune surveillance and attack (10). Substantial evidence shows that the tumor microenvironment (TME) is crucial in tumor cell invasion and metastasis (11, 12). Chemokines, serving as key messengers for intercellular communication, may significantly influence the TME. Among the various chemokines, CCL2 (MCP-1) is involved in several key processes, including the recruitment of immunosuppressive cells, promotion of angiogenesis, and direct enhancement of tumor cell invasiveness (13, 14). While CCL2 is recognized as a significant pro- or anti-tumor factor in many solid tumors (15, 16), its specific role in the progression of OSCC, particularly in studies at both tissue and serum levels, remains underexplored. This study conducted a clinical investigation integrating histological and serological analyses. We compared CCL2 expression in oral squamous cell carcinoma (OSCC) tissues and adjacent non-cancerous tissues using immunohistochemistry. Additionally, we monitored changes in peripheral serum CCL2 concentration in patients during the peri-operative period using ELISA. By correlating these findings with patients’ clinicopathological and follow-up data, we aimed to elucidate the expression pattern of CCL2 in OSCC and assess its potential as a prognostic biomarker or therapeutic target.

## 2 Materials and methods

### 2.1 Patients and tissue specimens

This study involved patients histopathologically diagnosed with oral squamous cell carcinoma (OSCC) at Hunan Cancer Hospital between June 2018 and January 2019. Prior to enrollment, all patients provided informed consent for the use of their clinical data in this research. The study included 119 patients, comprising 105 males and 14 females, with a median age of 55 years (range: 31–74 years). All 119 patients underwent R0 resection. During the operation, the negative resection margins were confirmed through rapid frozen pathology. Among them, 43 patients had lymphatic vessel involvement. Exclusion criteria were a history of previous malignancies, prior radiotherapy or chemotherapy, severe systemic infections within the past 3 months (excluding mild infections at the primary site), a history of major trauma or surgery, and recent use of vasoactive drugs. Tumor recurrence and metastasis were assessed through physical examinations, imaging evaluations, surgical records, and postoperative pathological examinations.

### 2.2 Immunohistochemistry

To assess CCL2 protein expression in patient tissues, we conducted immunohistochemical analysis on oral squamous cell carcinoma samples and adjacent normal tissue from 97 patients. Initially, we considered 119 patients, but some paracancerous tissues from postoperative paraffin specimens did not meet the criteria, leading to their exclusion. Thus, we selected 97 satisfactory cancer and adjacent tissue samples for comparison. Pathologists confirmed the tissue properties and section locations of both the carcinoma and normal epithelial tissues, all derived from postoperative paraffin specimens. Following established protocols (17), we dewaxed, hydrated, and retrieved antigens from formalin-fixed, paraffin-embedded sections. These sections were then incubated overnight at 4 °C with a rabbit polyclonal antibody against CCL2 (Catalog number: ab9669, Abcam, USA). For color development, we employed the PowerVision™ two-step staining kit (Catalog number: PV-6001, Beijing Zhongshan Golden Bridge Biotechnology Co., Ltd., China), and counterstained the nuclei with hematoxylin. Phosphate-buffered saline served as the negative control, replacing the primary antibody.

All the slices were independently evaluated by two pathologists who were unaware of the patients’ clinical and pathological information. The expression level of CCL2 was scored based on the staining intensity and the proportion of positive cells. The staining intensity was classified as: 0 (no staining), 1 (weak), 2 (moderate), 3 (strong); the proportion of positive cells was classified as: 0 (<5%), 1 (5%–25%), 2 (26%–50%), 3 (51%–75%), 4 (>75%). The final score was the product of the intensity score and the proportion score, ranging from 0 to 12 points. Based on the final score, the patients were divided into two groups: the low-expression group (0–3 points) and the high-expression group (4–12 points).

### 2.3 Enzyme-linked immunosorbent assay

To assess CCL2 secretion levels in the tumor microenvironment of patients, we collected peripheral blood serum samples from 119 patients both before surgery and on the 7th day post-surgery. Based on the changes of CCL2 in the patient’s peripheral blood, the trend of CCL2 in the tumor microenvironment can be reflected. We measured CCL2 concentrations using an enzyme-linked immunosorbent assay (ELISA), following the human MCP-1/CCL2 ELISA kit instructions from RayBiotech, USA (18). The procedure involved adding standards and samples to pre-coated plate wells, followed by biotin-labeled antibodies and horseradish peroxidase-labeled streptavidin. After developing color with a TMB substrate, we read the absorbance at 450 nm using a microplate reader. SigmaPlot 12.0 software was utilized to create the standard curve and determine CCL2 concentrations in the samples.

### 2.4 Follow-up

A total of 119 patients were regularly monitored after surgery, with a median follow-up period of 60 months (range: 6–60 months). Four patients were lost to follow-up due to changes in contact information or other reasons. Tumor recurrence and metastasis were diagnosed through clinical examination, imaging evaluation, and pathological examination. Overall survival was defined as the time from surgery to death caused by the disease, while disease-free survival was defined as the time from surgery to tumor recurrence or death. Non-tumor-related deaths were considered lost to follow-up in the survival analysis.

### 2.5 Statistical analysis

All statistical analyses utilized SPSS 22.0 software. Measurement data following a normal distribution were expressed as mean ± standard deviation, with comparisons before and after surgery conducted using the paired-sample t-test. Categorical variables were presented as frequencies and percentages, and comparisons between groups employed the chi-square test. The Kaplan–Meier method was used to construct the survival curve. All statistical tests were two-sided, with a P-value < 0.05 deemed statistically significant.

## 3 Results

### 3.1 Expression differences of CCL2 between oral squamous cell carcinoma and adjacent normal tissues

**Figure 1:**
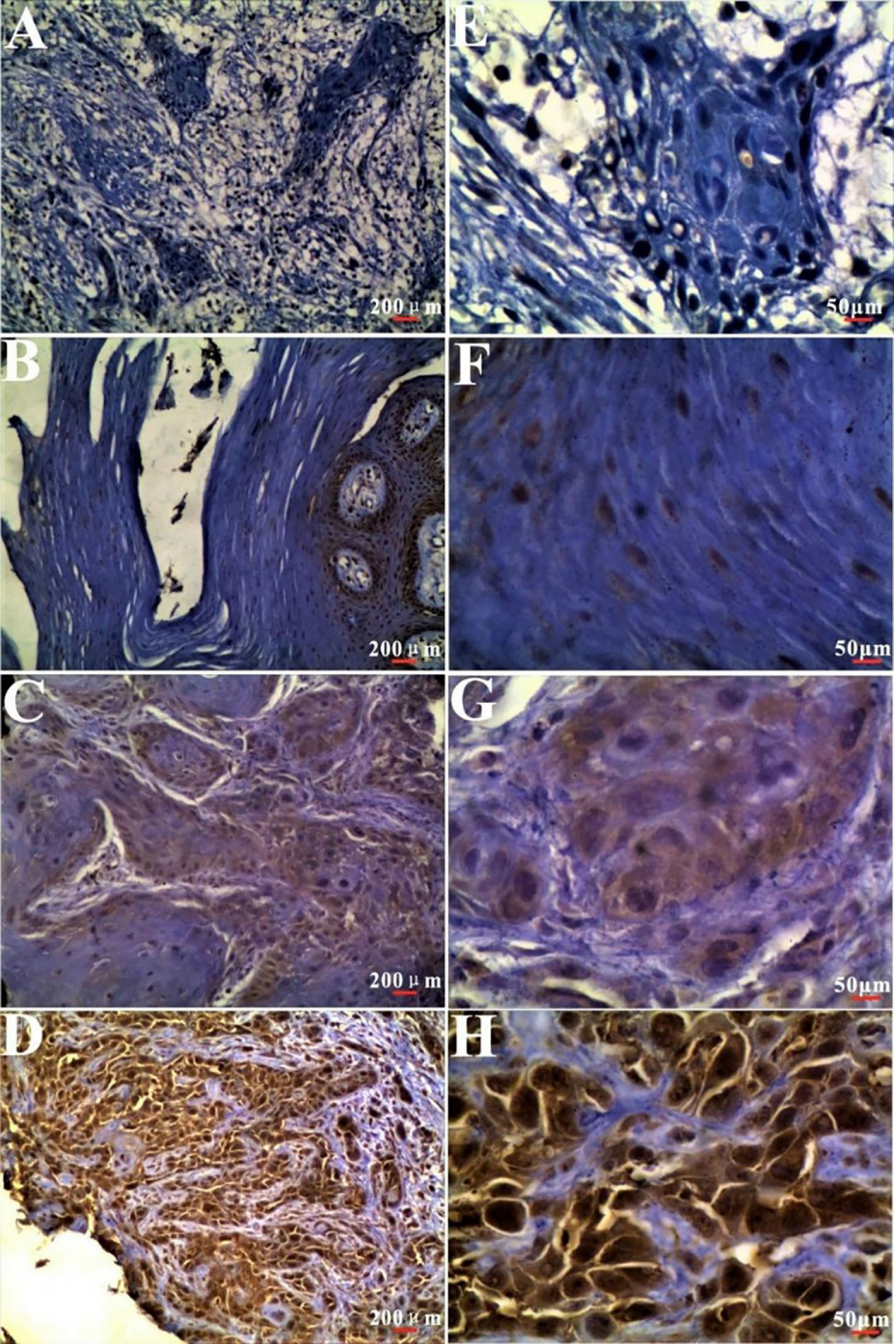
Immunohistochemical of Expression of CCL2 in oral squamous cell carcinoma and adjacent tissues CCL2 immunostaining was predominantly localized in the cytoplasm, with weaker expression observed in the nucleus. Brownish yellow deposits indicate positive staining. (A, E) Negative control. (B, F) CCL2 expression in adjacent normal tissues. (C, G) Weak positive CCL2 expression in OSCC tissues. (D, H) Strong positive CCL2 expression in OSCC tissues. (A-D) Original magnification, 100×. (E-H) Original magnification, 400×.

Immunohistochemical analysis showed that the CCL2 protein was predominantly localized in the cytoplasm (**Figures 1A–H**). In a study of 97 cases of oral squamous cell carcinoma and their adjacent normal tissues, cancer tissues demonstrated significantly higher CCL2 expression compared to the adjacent epithelial tissues, which exhibited low expression (**Table 1**). This difference in expression between the two groups was statistically significant (P < 0.05) (**Table 2**).

**Table 1:** Immunohistochemical scores of CCL2 expression in oral squamous cell carcinoma (OSCC) and matched adjacent normal tissues.

Group/score	1	2	4	6	8	9	12
OSCC	7	13	34	22	10	9	2
Adjacent Tissue	72	14	9	2	0	0	0

**Table 2:** The immunohistochemical expression differences of CCL2 in OSCC and adjacent tissue.

Group	No. of patients	CCL2 expression			
		High	Low	χ² value	P value
OSCC	97	77	20	90.594	**0.001**
Adjacent Tissue	97	11	86		

* P < 0.05 was considered to be statistical significance.

P-values in bold were statistically significant.

### 3.2 Correlation between CCL2 expression and clinical features, pathology, and prognosis

**Figure 2:**
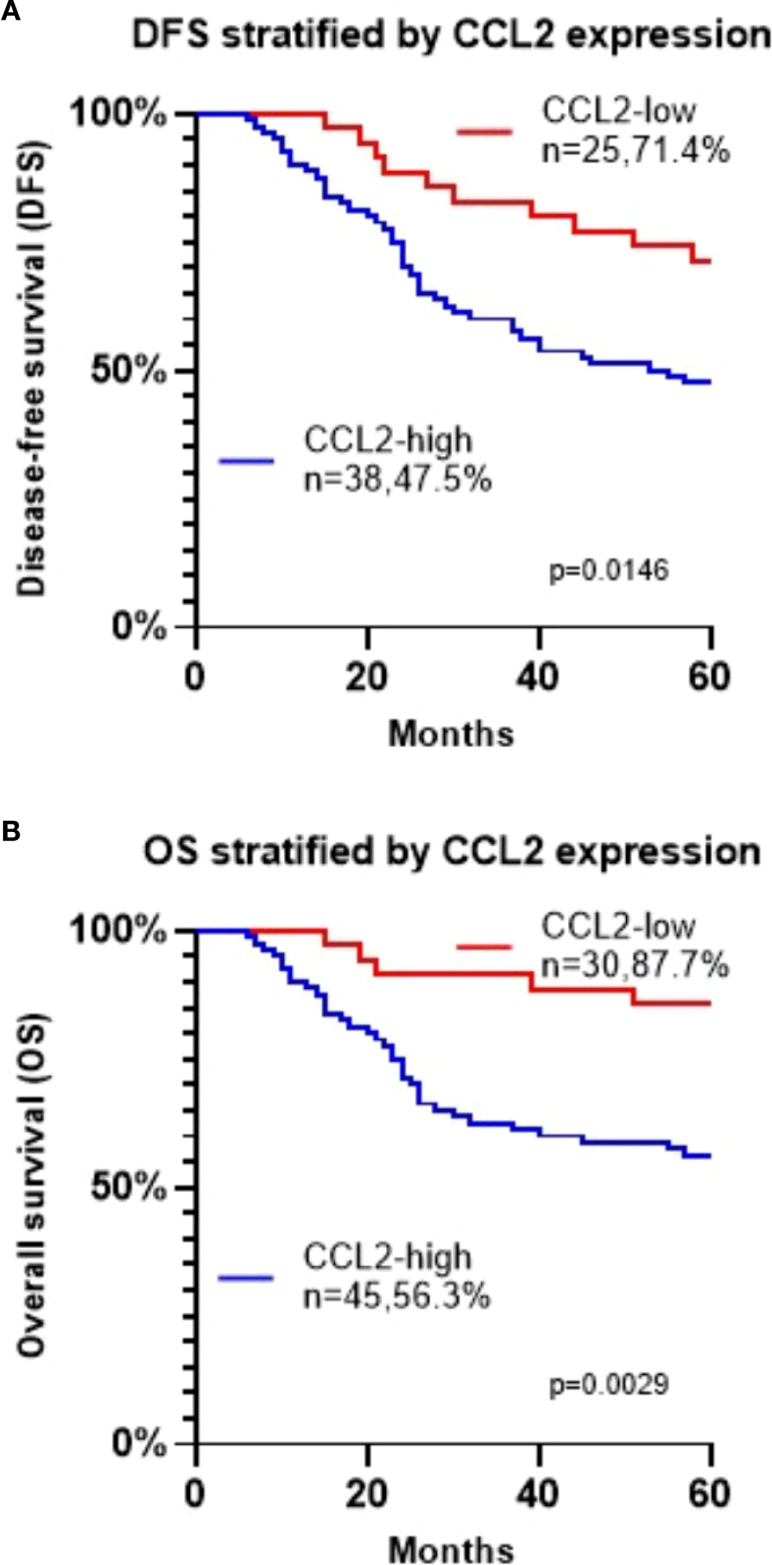
**(A)** Kaplan-Meier survival analysis of disease-free survival (DFS) based on CCL2 expression in patients with oral squamous cell carcinoma. **(B)** Kaplan-Meier survival analysis of over survival (OS) based on CCL2 expression in patients with oral squamous cell carcinoma.

To assess the relationship between varying CCL2 expressions and clinical and pathological features in oral squamous cell carcinoma, we performed a stratified analysis of patients. The chi-square test revealed that high CCL2 expression significantly correlated with advanced T stage, later clinical stage, poor differentiation, lymph node metastasis, recurrence, and mortality (**Table 3**, P < 0.05). However, it showed no significant association with gender or age (**Table 3**, P > 0.05). Kaplan-Meier survival analysis further demonstrated that patients with elevated CCL2 expression experienced significantly reduced disease-free survival (DFS) and overall survival (OS) (P < 0.05, **Figures 2A, B**).

**Table 3:** Correlation analysis of CCL2 expressions with clinical and pathological features.

Group	No. of patients	CCL2 expression			
		High	Low	χ² value	P value
T Stage					
T 1-2	82	50	32	9.618	**0.002**
T 3-4	37	33	4		
N Stage					
N 0	67	38	29	13.316	**0.001**
N 1	25	20	5		
N 2	27	25	2		
Clinical Stage					
Stage I-II	49	24	25	17.028	**0.001**
Stage III-IV	70	59	11		
Differentiation					
Well differentiated	91	59	32	4.424	**0.035**
Moderately/Poorly differentiated	28	24	4		
Gender					
Male	105	66	39	0.011	0.917
Female	14	9	5		
Age					
≥56 years	63	44	19	0.001	0.981
<56 years	56	39	17		
Recurrence					
Yes	52	42	10	5.628	**0.018**
No	63	38	25		
Mortality					
Yes	40	35	5	9.318	**0.002**
No	75	45	30		

* P < 0.05 was considered to be statistical significance.

P-values in bold were statistically significant.

a Four patients lost to follow-up because of telephone number changes or home moving.

### 3.3 Alterations in CCL2 expression within the cellular microenvironment of the *in vitro* model

**Figure 3:**
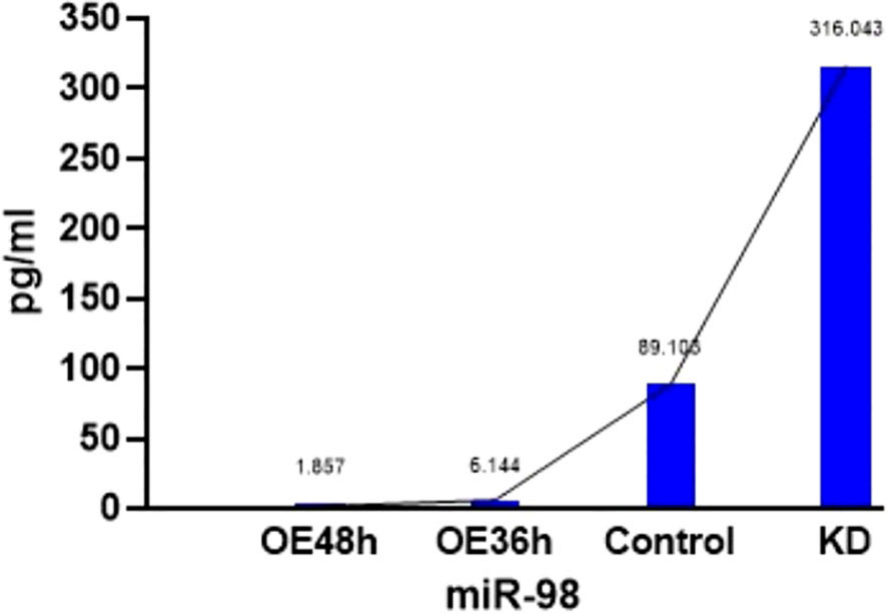
CCL2 concentration in culture supernatant of FADU cells with modulated miR-98 expression.

To investigate potential changes in CCL2 within the tumor microenvironment, we performed functional validation using the FADU cell line. We manipulated the expression level of miR-98, a known OSCC metastasis suppressor, and observed its significant impact on cell invasion and CCL2 secretion. When miR-98 was silenced, enhancing invasion ability, CCL2 secretion in the culture supernatant increased more than threefold compared to the control group. Conversely, in cells with overexpressed miR-98, which exhibited reduced invasion ability, CCL2 secretion significantly decreased (**Figure 3**). These findings indicate that elevated CCL2 secretion in the cell microenvironment correlates with the strong invasive phenotype of OSCC cells.

### 3.4 Correlation between perioperative serum CCL2 levels and clinical, pathological features, and prognosis

**Figure 4:**
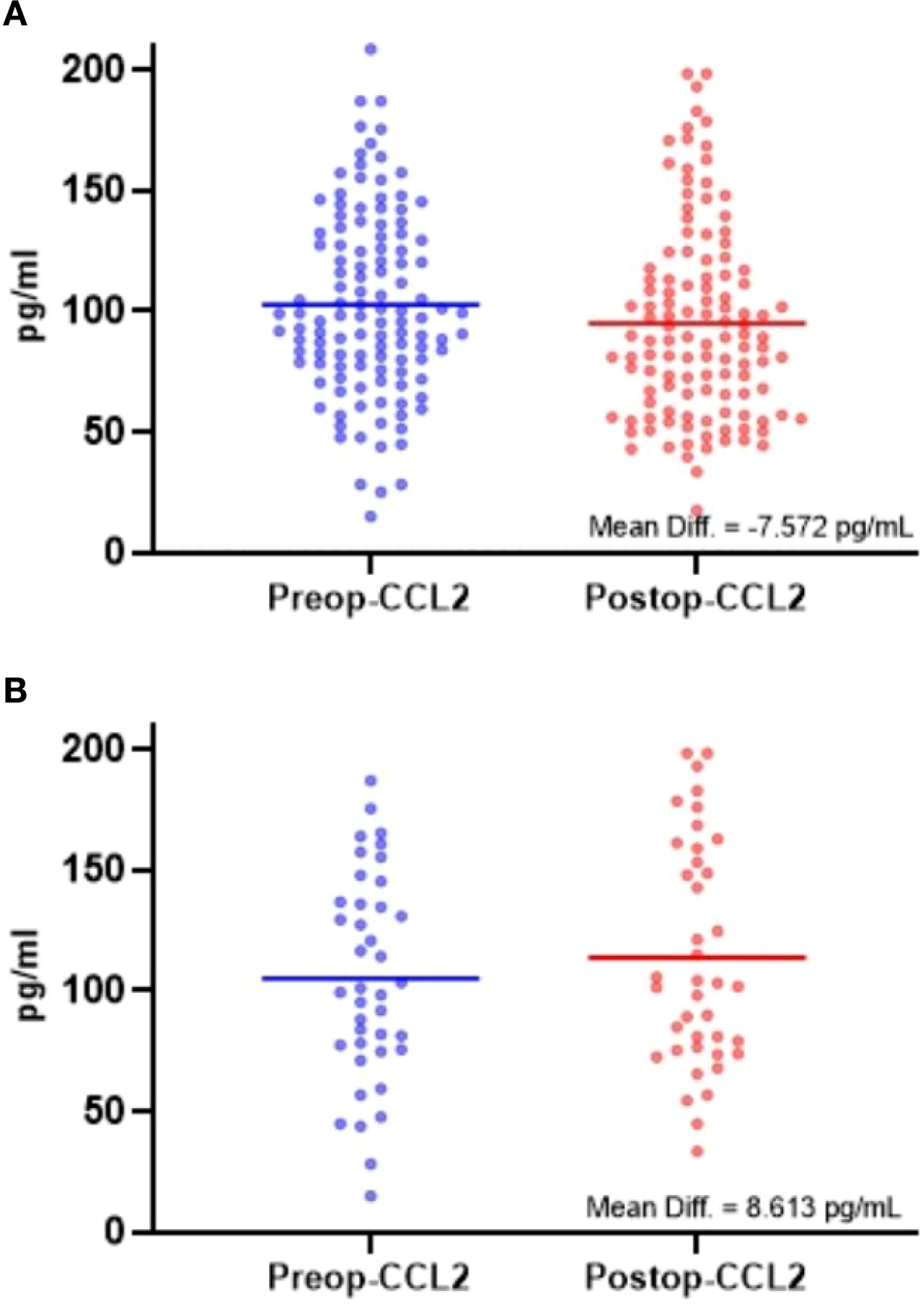
**(a)** Comparison of serum CCL2 concentrations in 119 OSCC patients before and after surgery (paired t-test). Data are presented as mean ± SD. The mean serum CCL2 concentration decreased from 102.58003 pg/mL preoperatively to 95.008 pg/mL postoperatively, with a mean difference of 7.571 pg/mL (paired t-test, P =0.0042). **(b)** Comparison of serum CCL2 concentrations in 40 deceased OSCC patients before and after surgery (paired t-test). Data are presented as mean ± SD. The mean serum CCL2 concentration increased from 105.047 pg/mL preoperatively to 113.6606 pg/mL postoperatively, with a mean difference of -8.6126 pg/mL (paired t-test, P = 0.034).

Analyzing peri-operative serum CCL2 concentrations in 119 oral squamous cell carcinoma patients revealed a significant postoperative decrease in CCL2 levels (**Table 4**; **Figure 4a**, P < 0.05). Subgroup analysis indicated this decline was particularly pronounced in patients with early-stage tumors (T1-2, clinical stage I-II), no lymph node metastasis, no recurrence, and those who survived (**Table 4**, P < 0.05). Conversely, in deceased patients(those who died due to tumor recurrence), postoperative CCL2 levels increased (**Table 4**; **Figure 4b**, P < 0.05). No significant changes in CCL2 levels were observed in patients with advanced-stage tumors (T3-4, clinical stage III-IV) or those with recurrent disease, and there were no statistical differences across different age and gender groups (**Table 4**, P > 0.05).

**Table 4:** Correlation analysis of changes in CCL2 before and after the operation with clinical and pathological features.

Group	No. of patients	Preop mean	Postop mean	Mean diff.	t value	P value
		(pg/ml)	(pg/ml)	(pg/ml)		
All patients	119	102.580	95.008	7.571	2.953	**0.004**
T stage						
T 1-2	82	101.166	88.284	12.881	4.759	**0.001**
T 3-4	37	105.713	109.909	-4.195	-0.805	0.426
N stage						
N 0	67	100.137	92.436	7.701	2.222	**0.030**
N +	52	105.727	98.322	7.404	1.926	0.060
N 1	25	100.317	84.918	15.398	2.760	**0.011**
N 2	27	100.736	100.734	0.002	0.000	1.000
Clinical stage						
Stage I-II	49	101.340	88.781	12.559	3.511	**0.001**
Stage III-IV	70	103.447	99.367	4.080	1.156	0.252
Differentiation						
Well differentiated	91	99.194	88.943	10.251	3.818	**0.001**
Moderately/Poorly differentiated	28	113.583	114.721	-1.137	-0.179	0.859
Recurrence						
Yes	52	103.119	107.911	-4.792	-1.229	0.225
No	63	103.045	84.857	18.188	6.182	**0.001**
Mortality						
Yes	40	105.047	113.660	-8.612	-2.200	**0.034**
No	75	102.029	85.480	16.548	5.583	**0.001**

* P < 0.05 was considered to be statistical significance.

P-values in bold were statistically significant.

a Four patients lost to follow-up because of telephone number changes or home moving.

Further analysis revealed that CCL2 levels decreased post-surgery in 79.36% (50/63) of non-recurrent patients and 78.67% (59/75) of surviving patients. Conversely, CCL2 increased in 55.76% (29/52) of recurrent patients and 65.00% (26/40) of deceased patients. The chi-square test confirmed a significant association between the trend in CCL2 levels and both tumor recurrence and patient mortality (P < 0.05) (**Table 5**).

**Table 5:** Correlation analysis of CCL2 changes with clinical and pathological features.

Group	No. of Patients	CCL2 Change			
		Postop-decrease	Postop-increase	χ² Value	P Value
Recurrence					
Yes	52	21	31	21.836	**0.001**
No	63	52	11		
Mortality					
Yes	40	12	28	29.651	**0.001**
No	75	61	14		

* P < 0.05 was considered to be statistical significance.

P-values in bold were statistically significant.

a Four patients lost to follow-up because of telephone number changes or home moving.

**Figure 5:**
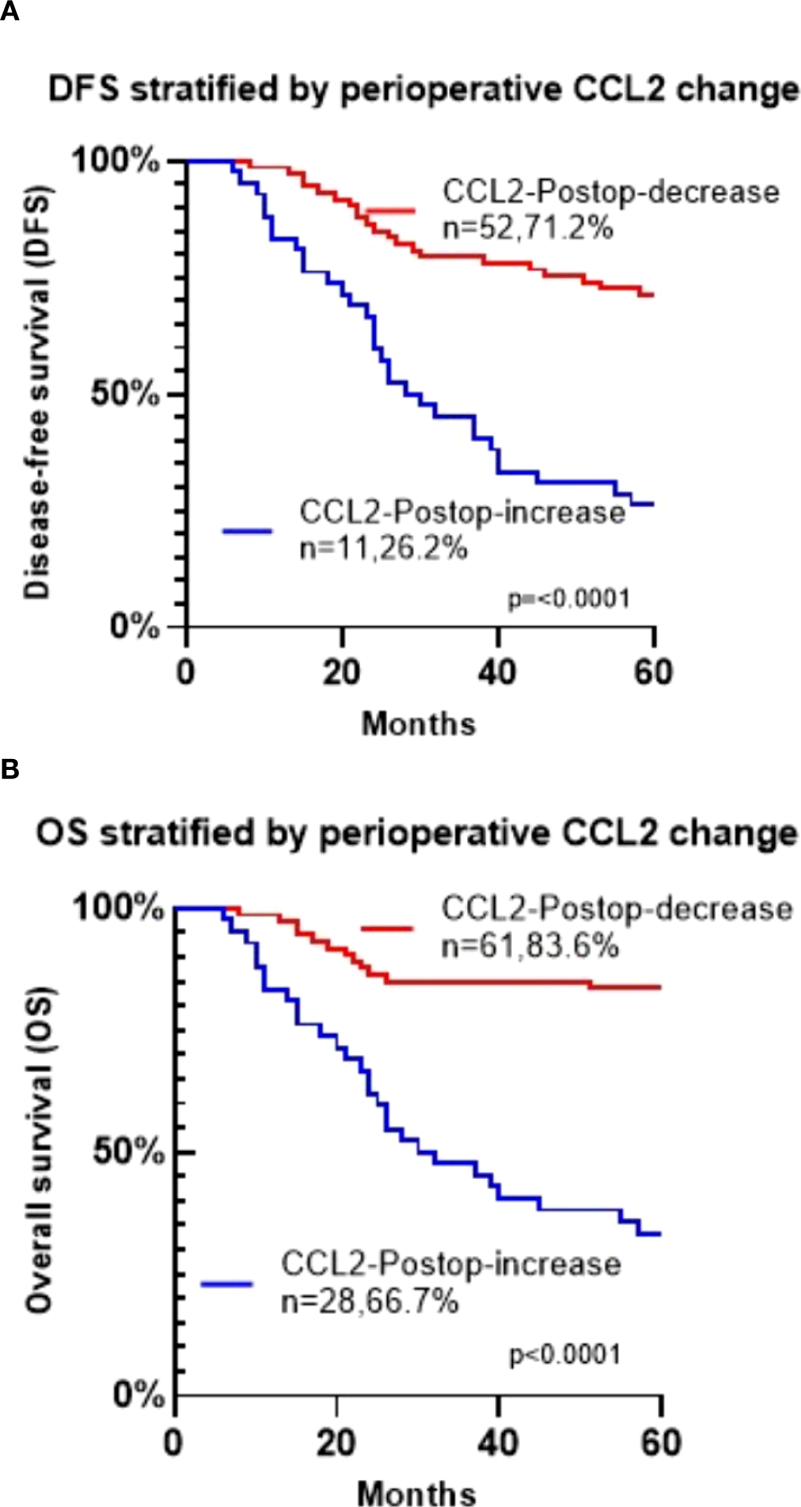
**(A)** Kaplan-Meier survival analysis of disease-free survival (DFS) in patients with oral squamous cell carcinoma based on changes in CCL2. **(B)** Kaplan-Meier survival analysis of overall survival (OS) in patients with oral squamous cell carcinoma based on changes in CCL2.

**Figure 6:**
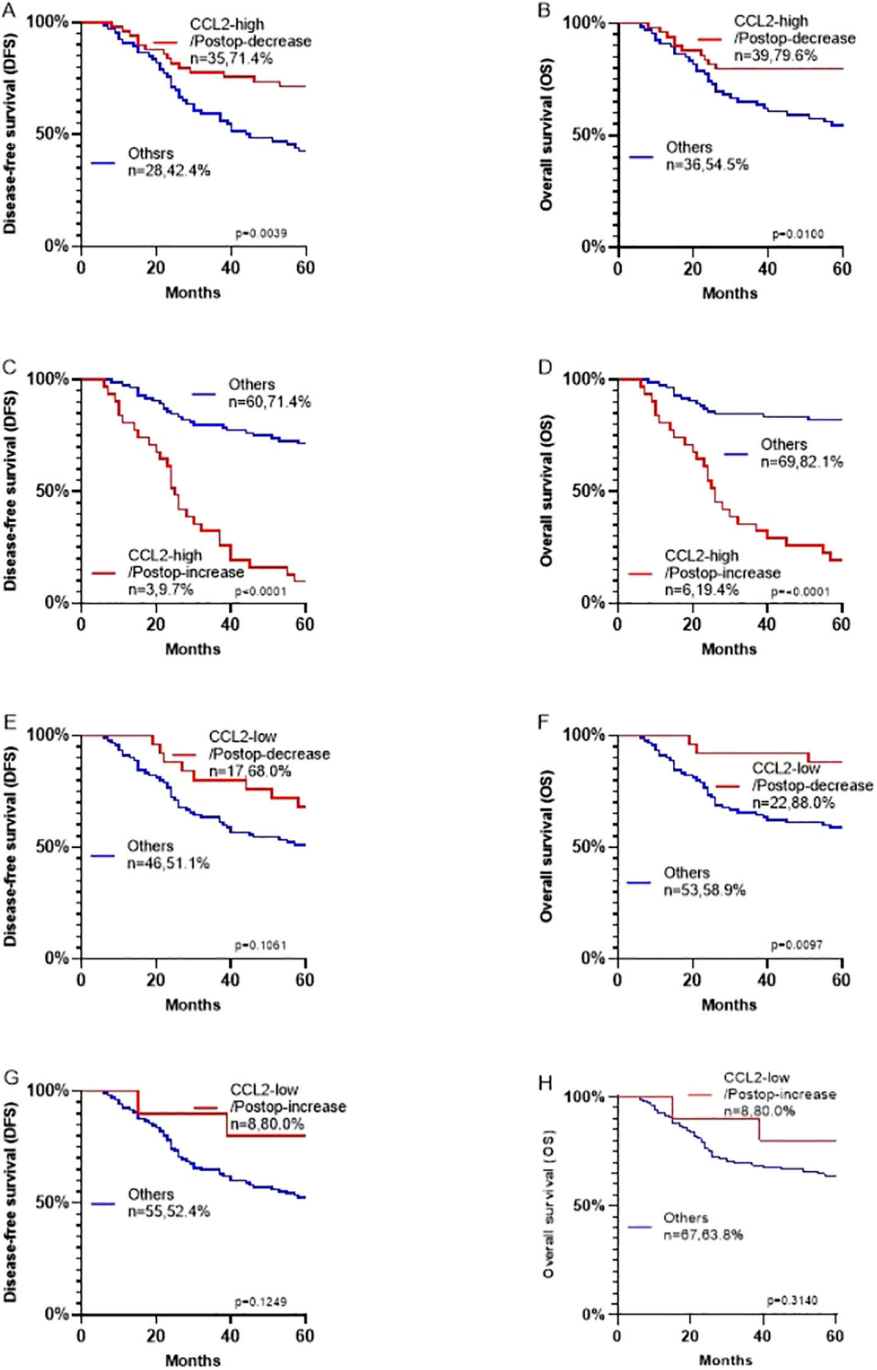
**(A–H)** Kaplan-Meier survival analysis based on the expression and changes of CCL2 for disease-free survival (DFS) and overall survival (OS) of patients with oral squamous cell carcinoma.

Survival analysis showed that a postoperative decrease in CCL2 correlated with longer disease-free and overall survival (**Figures 5A, B**, P < 0.05). Importantly, the combined analysis demonstrated that patients with high CCL2 expression in tumor tissues and a postoperative decrease in serum CCL2 had a more favorable prognosis (**Figures 6A, B**, P < 0.05). Conversely, those with high tissue expression and a postoperative increase in serum CCL2 experienced the poorest prognosis (**Figures 6C, D**, P < 0.05).

## 4 Discussion

The chemokine CCL2 (MCP-1), a key member of the CC chemokine family, plays diverse roles in tumorigenesis and development (19, 20). It is involved in remodeling the tumor microenvironment, inducing immune suppression, and promoting tumor invasion and metastasis (12). CCL2 recruits immunosuppressive cells, such as tumor-associated macrophages (TAMs) and myeloid-derived suppressor cells (MDSCs), to the tumor site (21). This recruitment inhibits effector T cell functions and creates an immune-tolerant microenvironment, aiding tumor cells in evading immune surveillance. Additionally, CCL2 binds to the CCR2 receptor on tumor cells, activating signaling pathways like PI3K/Akt and MAPK. This activation promotes tumor cell proliferation, epithelial-mesenchymal transition (EMT), and invasion (14). Furthermore, CCL2 induces vascular endothelial cell migration and new blood vessel formation, supporting tumor growth and metastasis (22). Previous studies have confirmed CCL2’s pro-tumor or anti-tumor roles in various cancers, including breast, non-small cell lung, colorectal, pancreatic, and ovarian cancers (23). These findings suggest its effects are somewhat universal across different tumor types, prompting further exploration of CCL2’s specific role in oral squamous cell carcinoma (OSCC).

In this study, we compared 97 pairs of oral squamous cell carcinoma (OSCC) tissues with adjacent normal tissues and discovered that CCL2 was significantly highly expressed in cancer tissues. This finding aligns with reports from other tumors, reinforcing CCL2’s potential role in OSCC development (24). The literature indicates that CCL2 can either promote or inhibit various malignancies (23). Our data revealed that high CCL2 expression in OSCC correlated with increased local tumor invasion, higher lymph node metastasis rates, advanced clinical stages, poor tumor differentiation, patient recurrence, and reduced overall survival.

In this study, we found no significant correlation between CCL2 expression and gender. This lack of correlation may be due to statistical bias, as female patients comprised only 11.76% of the sample. Previous research has indicated that CCL2 secretion varies between genders in tumors associated with sex hormones. However, in head and neck squamous cell carcinoma, no gender differences in CCL2 expression were observed. Patient ages ranged from 31 to 74 years, with a median age of 55 years, aligning with the high-incidence age range for OSCC (25). Consequently, we could not draw a definitive conclusion about the relationship between CCL2 expression and age.

We discovered that CCL2 expression levels correlated with the occurrence of lymph node metastasis, though not with its severity. We propose that CCL2, as a secreted protein, likely does not directly facilitate tumor cell metastasis. Instead, it functions as an initiator within the tumor microenvironment, influencing biological processes related to metastasis. Presently, the alterations of CCL2 in the OSCC cell microenvironment are not well understood and warrant further investigation.

Previous studies have identified miR-98 as a tumor suppressor gene in oral cancer cells (26).Oral squamous cell carcinoma cells exhibit varying levels of invasiveness depending on miR-98 expression (27). We previously speculated that altering miR-98 expression to modify tumor cell invasiveness would impact CCL2 levels in the culture supernatant of these cells. Our experimental results confirmed that cells with increased invasiveness had higher CCL2 content, whereas cells with reduced invasiveness showed a marked decrease in CCL2 secretion. This suggests a potential link between CCL2 changes in the tumor microenvironment and tumor invasiveness.

We used ELISA to measure CCL2 levels in the peripheral serum of perioperative patients, aiming to reflect changes in the local microenvironment. Our findings showed a significant decrease in postoperative CCL2 concentrations compared to pre-surgery levels (P < 0.05), suggesting that tumor resection effectively removed the primary CCL2 source. This change in concentration may indicate the completeness of surgical resection. However, patients with T3–4 stage, moderately or poorly differentiated tumors, and stage III–IV did not exhibit a significant postoperative decrease in CCL2 levels. We speculate that this lack of change is due to factors such as high tumor burden, extensive infiltration of adjacent tissues from late-stage tumors, or a pronounced local inflammatory response.

After a 5-year follow-up, researchers observed an upward trend in postoperative serum CCL2 levels among patients who death (40 cases). Patients exhibiting high CCL2 expression in tumor tissues alongside elevated postoperative serum CCL2 levels had significantly shorter disease-free and overall survival rates (P < 0.05). This suggests a potential continuous secretion of CCL2 in these individuals. We hypothesize that this may result from the formation of a microenvironment with persistent CCL2 secretion. In such an environment, high CCL2 expression may promote the rapid growth and metastasis of residual cancer cells. Additionally, scattered tumor cells in the blood or lymphatic circulation might evade the immune system under conditions of immune suppression, continuously releasing CCL2. Elevated CCL2 levels can recruit more immunosuppressive cells to the tumor bed, lymphatic drainage areas, or around micrometastatic foci, exacerbating local immunosuppression and facilitating recurrence and metastasis. Conversely, patients with high tumor CCL2 expression but decreased postoperative serum CCL2 levels showed better prognoses. This indirectly suggests that the thoroughness of surgery is closely linked to the normalization of CCL2 levels in the microenvironment.

Due to constraints in follow-up duration, timing, and frequency of blood sampling, a parallel comparison was not possible. Currently, the existing data do not allow for quantification of CCL2 changes, necessitating further investigation in future studies.

In conclusion, the highly expressed CCL2 in oral squamous cell carcinoma strongly correlates with increased tumor invasiveness and poor prognosis. Persistently elevated CCL2 levels post-surgery suggest a high risk of recurrence, metastasis, and poor outcomes. Monitoring serum CCL2 levels dynamically may serve as a biomarker for assessing surgical efficacy and predicting recurrence risk. The CCL2/CCR2 signaling axis offers a promising research avenue for treating oral squamous cell carcinoma. However, further *in vivo* experiments are necessary to confirm the role of the CCL2/CCR2 axis in tumor progression and its therapeutic potential.

## Data Availability

The original contributions presented in the study are included in the article/Supplementary Material. Further inquiries can be directed to the corresponding author.
