# Correction: The prognostic significance of CCL2 in oral squamous cell carcinoma: insights from tissue expression and perioperative serum profiling

**DOI:** 10.3389/fonc.2026.1840750

**Published:** 2026-04-09

**Authors:** Senli Wen, Haolei Tan

**Affiliations:** 1 The First Department of Head and Neck Surgery, Hunan Cancer Hospital, the Affiliated Cancer Hospital of Xiangya School of Medicine, Central South University, Changsha, Hunan, China; 2 Department of Breast and Thyroid Surgery, The First Hospital of Changsha, the Affiliated Changsha Hospital of Xiangya School of Medicine, Central South University, Changsha, Hunan, China

**Keywords:** CCL2, lymph node metastasis, oral squamous cell carcinoma, perioperative monitoring, prognosis, serum biomarker

Affiliation 2 “Department of Breast and Thyroid Surgery, The First Hospital of Changsha, the Affiliated Changsha Hospital of Xiangya School of Medicine, Central South University, Changsha, Hunan, China” was omitted for author Senli Wen. This affiliation has now been added for author Senli Wen.

Affiliation 1 “The First Department of Head and Neck Surgery, Hunan Cancer Hospital, the Affiliated Cancer Hospital of Xiangya School of Medicine, Central South University, Changsha, China” was omitted for author Haolei Tan. This affiliation has now been added for author Haolei Tan.

Author Haolei Tan was erroneously assigned to Affiliation 2 “Department of Breast and Thyroid Surgery, The First Hospital of Changsha, the Affiliated Changsha Hospital of Xiangya School of Medicine, Central South University, Changsha, Hunan, China”. This affiliation has now been removed for author Haolei Tan.

The original version of this article has been updated.

